# Supplementary material for: Causal inference and cognitive-behavioral integration deficits drive stable variation in human punishment sensitivity
Source: Commun Psychol. 2025 Jul 9;3:103. doi: 10.1038/s44271-025-00284-9 (PMC12241639; doi:10.1038/s44271-025-00284-9)
Supplement: Supplementary file 2 — Supplemental Information [file 44271_2025_284_MOESM2_ESM.pdf]

Supplementary Information

Supplementary Figures S1 to S11

Supplementary Table S1

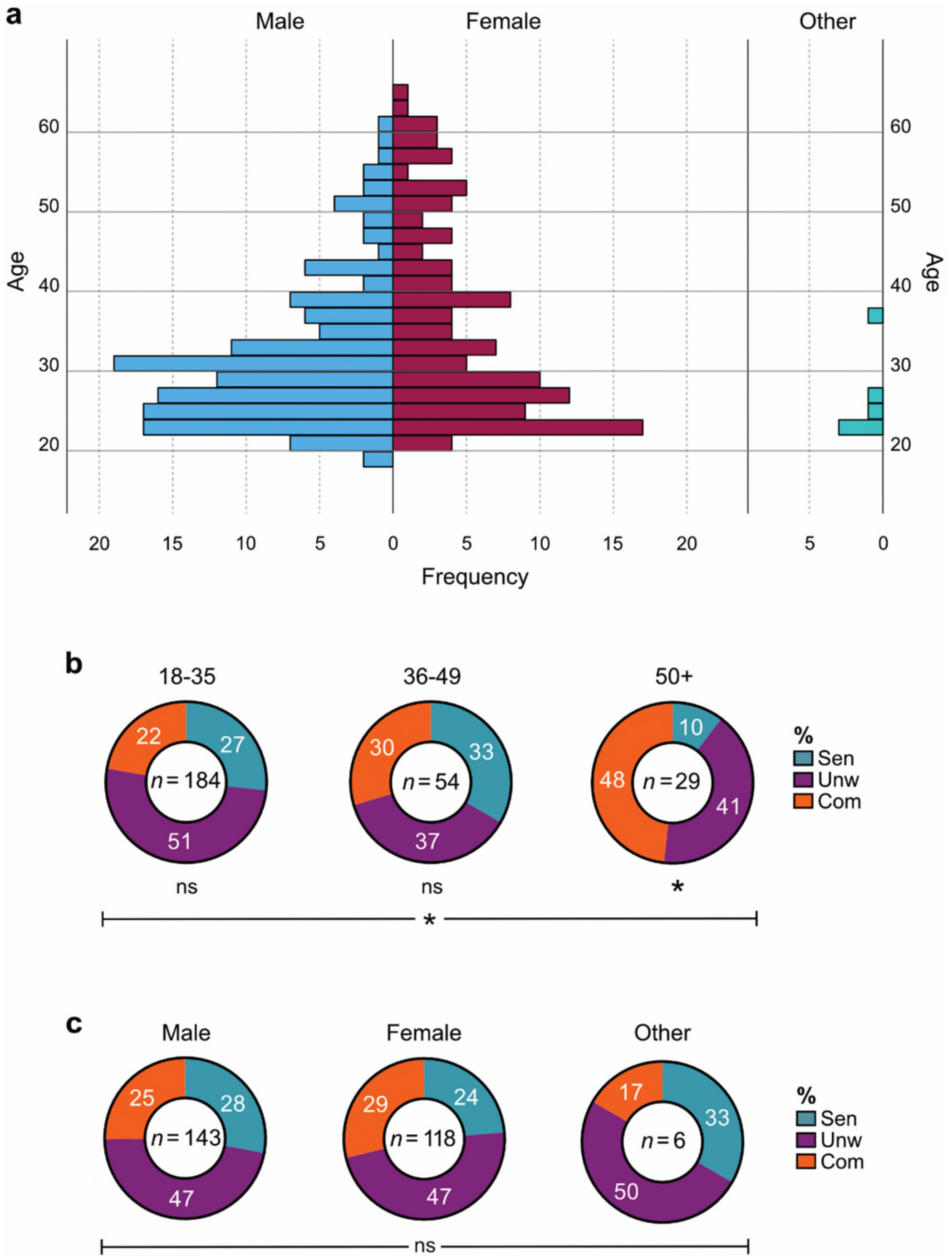

Figure S1. Demographics of participants and relationship to phenotypes. (A) Frequency distribution of participants by reported age and gender (*N*=267). **(B)** Phenotype composition by age bracket (18-35, 36-49, and 50+ years old). All phenotypes were observed in each age bracket, but composition differed across age brackets ($\chi^2_{(4)}$=12.42, *p*=.014); those in the oldest age bracket were significantly more likely to be Compulsive over Sensitive ($\chi^2_{(2)}$=8.113, *p*=.017). **(C)** Phenotype (Sensitive [Sen], Unaware [Unw], Compulsive [Com]) composition by gender. Genders did not significantly differ in their phenotype composition ($\chi^2_{(4)}$=1.127, *p*=.89). This held true when restricting analyses to those identifying as males and females ($\chi^2_{(2)}$= 0.771, *p*=.68).

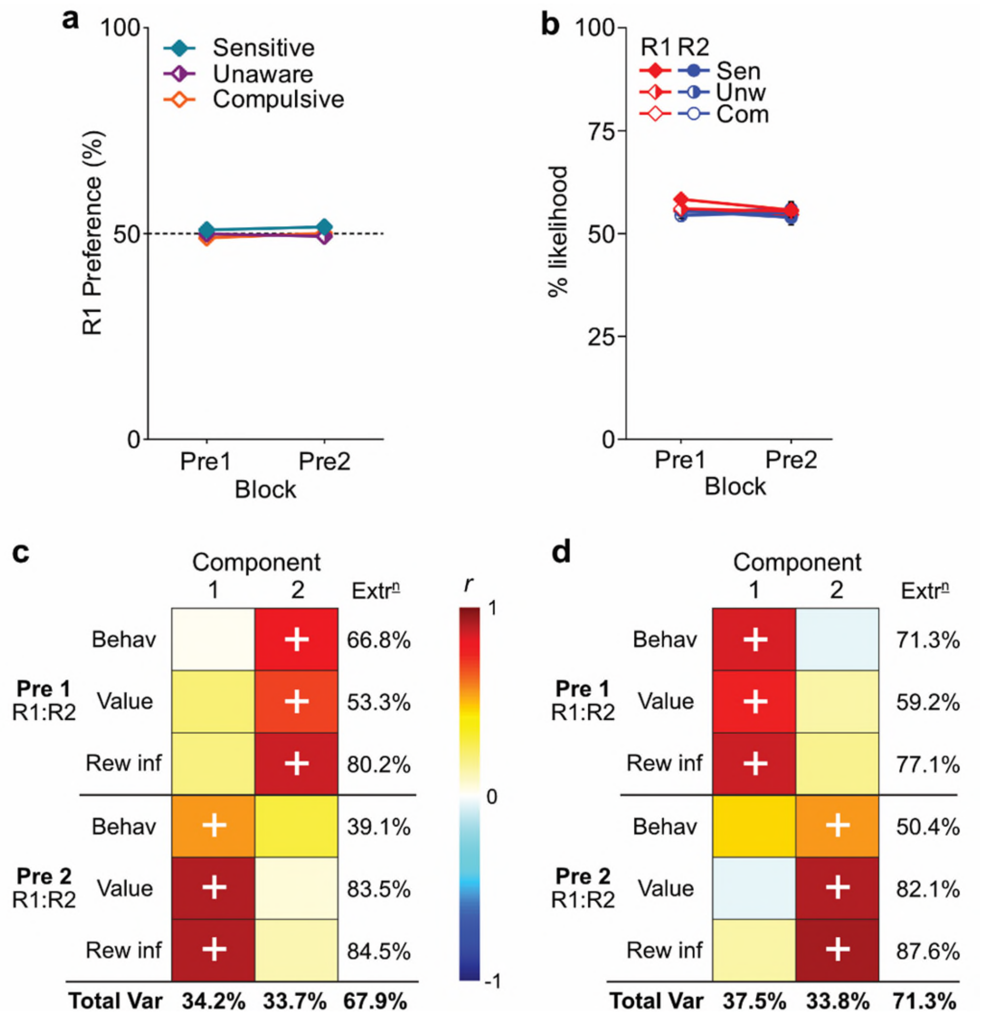

Figure S2. Responding and self-report measures during pre-punishment blocks. (A) Mean (±SEM) response preferences per cluster per pre-punishment block. All clusters responded equivalently on R1 vs R2 across the two pre-punishment blocks (Pre1, Pre2) (block: $F_{(1,264)}$=0.272, p=.602, η_p^2=.001 [90%CI: 0,.017]; cluster: $F_{(2,264)}$=1.065, p=.346, η_p^2=.008 [90%CI: 0,.030]; cluster*block: $F_{(2,264)}$=0.586, p=.557, η_p^2=.004 [90%CI: 0,.021]). **(B)** Mean (±SEM) Action→Reward inferences per cluster across blocks. Overall, all clusters accurately attributed equal reward to both actions across pre-punishment blocks (block: $F_{(1,264)}$=0.925, p=.337, η_p^2=.003 [90%CI: 0,.025]; action: $F_{(1,264)}$=0.941, p=.333, η_p^2=.004 [90%CI: 0,.025]; cluster: $F_{(2,264)}$=0.072, p=.931, η_p^2=.001 [90%CI: 0,.003]; cluster*action: $F_{(2,264)}$=0.016, p=.985, η_p^2<.001 [90%CI: 0,1]). **(C-D)** Principal Component Analysis of R1:R2 behavior (Behav), action valuation (Value), and reward inference (Rew inf) bias across pre-punishment blocks (Pre1, Pre2) for **(C)** all participants and **(D)** Compulsive cluster alone. White plus signs (+) indicate loadings above 0.5. In both cases, two components accounted for the majority of variance across measures: one component that accounted for variance in Pre1 measures, and another that accounted for variance in Pre2 measures. This indicates individual differences in response preference across blocks were related to individuals' perceptions of relative reward probability and action values. This indicates that participants, including Compulsives, were distinguishing between R1 and R2, even in pre-punishment. It is important to note that these are in fact spurious perceptions of biased reward probability; reward probability was 50% for both R1 and R2, and a reward was guaranteed

per 2 clicks on a planet (i.e., experienced reward probability never truly deviated from 50%). *N*=267 participants.

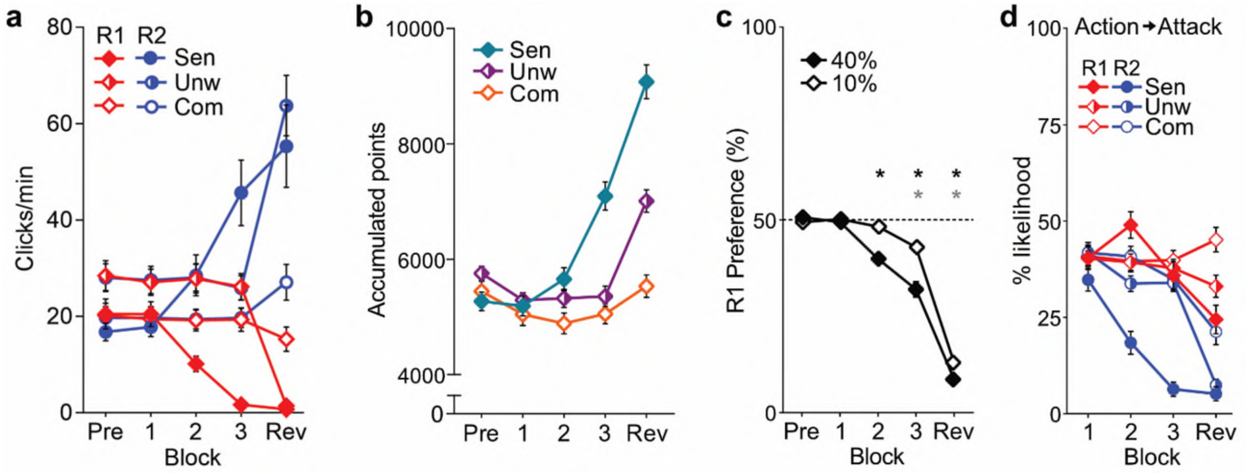

Figure S3. Additional task-related measures (initial test). (A) Mean (±SEM) response rates during non-CS periods for Sensitive (Sen), Unaware (Unw), and Compulsive (Com) clusters across blocks. Clusters did not notably differ in their overall click rates across pre-reveal blocks (cluster: $F_{(2,264)}$=3.317, p=.038, η_p^2=.025 [90%CI: .001,.059]; block*cluster: $F_{(2,264)}$=2.201, p=.113, η_p^2=.02 [90%CI: 0,.045]), indicating similar effort expenditure to gain points. Clusters primarily differed in their response strategy (action*cluster [pre-reveal]: $F_{(2,264)}$=26.82, p<.001, η_p^2=.169 [90%CI: .102,.232]); Sensitives reallocated efforts away from punished R1 to unpunished R2 (action*block: $F_{(1,69)}$=32.58, p<.001, η_p^2=.321 [90%CI: .174,.443]), whereas Unawares (action: $F_{(1,125)}$<.001, p=.994 [90%CI: 0,1]; action*block: $F_{(1,125)}$<.001, p=.988 [90%CI: 0,1]) and Compulsives (action: $F_{(1,70)}$=.007, p=.932 [90%CI: 0,.008]; action*block: $F_{(1,70)}$=.182, p=.671 [90%CI: 0,.053]) did not. Clusters also diverged following the reveal (action*cluster [reveal]: $F_{(2,264)}$=20.47, p<.001, η_p^2=.134 [90%CI: .073,.195]). Unawares strongly reallocated responding (action: $F_{(1,125)}$=97.13, p<.001, η_p^2=.437 [90%CI: .330,.522]; action*block: $F_{(1,125)}$=88.52, p<.001, η_p^2=.415 [90%CI: .306,.502]), while Compulsives exhibited a more modest shift (action: $F_{(1,70)}$=20.915, p<.001, η_p^2=.23 [90%CI: .098,.357]; action*block: $F_{(1,70)}$=17.01, p<.001, η_p^2=.196 [90%CI: .072,.322]). **(B)** Mean (±SEM) points accumulated per cluster across blocks. Clusters differed in point accumulation across pre-reveal punishment (block*cluster: $F_{(2,264)}$=78.618, p<.001, η_p^2=.229 [90%CI: .297,.436]); only Sensitives gained points (block: $F_{(1,69)}$=70.01, p<.001, η_p^2=.504 [90%CI: .360,.602]), whereas Unawares (block [pre-reveal]: $F_{(1,125)}$=3.884, p=.051,η_p^2=.003 [90%CI: 0,.094]) and Compulsives (block [pre-reveal]: $F_{(1,70)}$=3.981, p=.050,η_p^2=.054 [90%CI: <.001,.157]) tended to lose points. **(C)** Mean (±SEM) R1 preferences per probability group. Low probability (but high severity) punishment caused less punishment avoidance than high probability (low severity) punishment (group [pre-reveal]: $F_{(1,265)}$=17.18, p<.001, η_p^2=.061 [90%CI: .022,.112]). *p<.05 one sample t-test vs. 50% [no R1:R2 bias]). There was no effect of punishment probability after controlling for cluster (Pre-reveal: $F_{(1,261)}$=2.226, p=.137 [90%CI: 0,.036]; Reveal: $F_{(1,261)}$=.002, p=.967 [90%CI: 0,1]). **(D)** Mean (±SEM) Action→Attack inferences per cluster across blocks. Clusters differed in their attribution of attacks to R1 vs R2 prior to reveal (action*cluster: $F_{(2,264)}$=38.921, p<.001, η_p^2=.228 [90%CI: .155,.293]); only Sensitives reliably attributed attacks to R1 over R2 (action [Sensitive]: $F_{(1,69)}$=94.57, p<.001, η_p^2=.578 [90%CI: .446,.663]; [Unaware]: $F_{(1,125)}$=3.371, p=.069, η_p^2=.026 [90%CI: 0,.087]; [Compulsive]: $F_{(1,70)}$=0.371, p=.544, η_p^2=.005 [90%CI: 0,.065]). Unawares (action: $F_{(1,125)}$=57.75, p<.001, η_p^2=.316 [90%CI: .207,.411]; action*block: $F_{(1,125)}$=32.81, p<.001, η_p^2=.208 [90%CI: .110,.305]) and Compulsives (action: $F_{(1, 70)}$=24.92, p<.001,η_p^2=.263 [90%CI: .124,.388]; action*block: $F_{(1, 70)}$=22.35, p<.001,η_p^2=.242 [90%CI: .107,.369]) exhibited greater Action-Attack awareness following the reveal. N=267 participants.

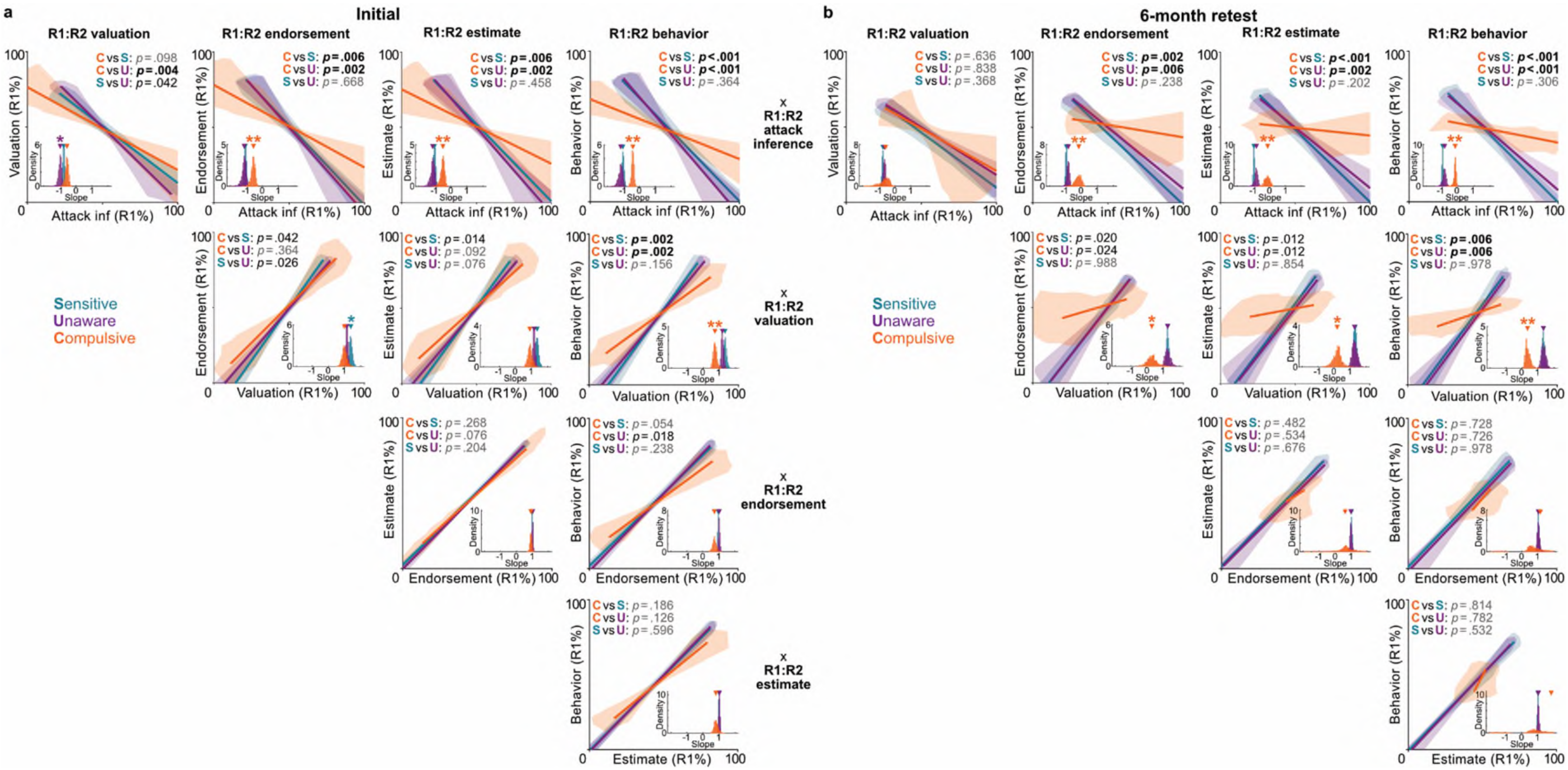

Figure S4. Pairwise trajectories between R1:R2 measures. Relationships between R1:R2 attack inferences, R1:R2 valuations, R1:R2 endorsements, R1:R2 estimates, and R1:R2 behavior, as determined via singular value decomposition (per cluster) for **(A)** initial test (N=267) and **(B)** retest (N=128) data. *Main plots*: colored lines indicate the identified true-score relationship between pairs of measures; shaded regions indicate extent of 1000 bootstrapped true-score relationship estimates (99.9% confidence region). C (*orange*) = Compulsive; S (*teal*) = Sensitive; U (*purple*) = Unaware. *Inset histograms*: Distribution of bootstrapped slope coefficients. Arrow indicates identified true-score coefficient. * p<.05; ** p<.01 vs. other clusters.

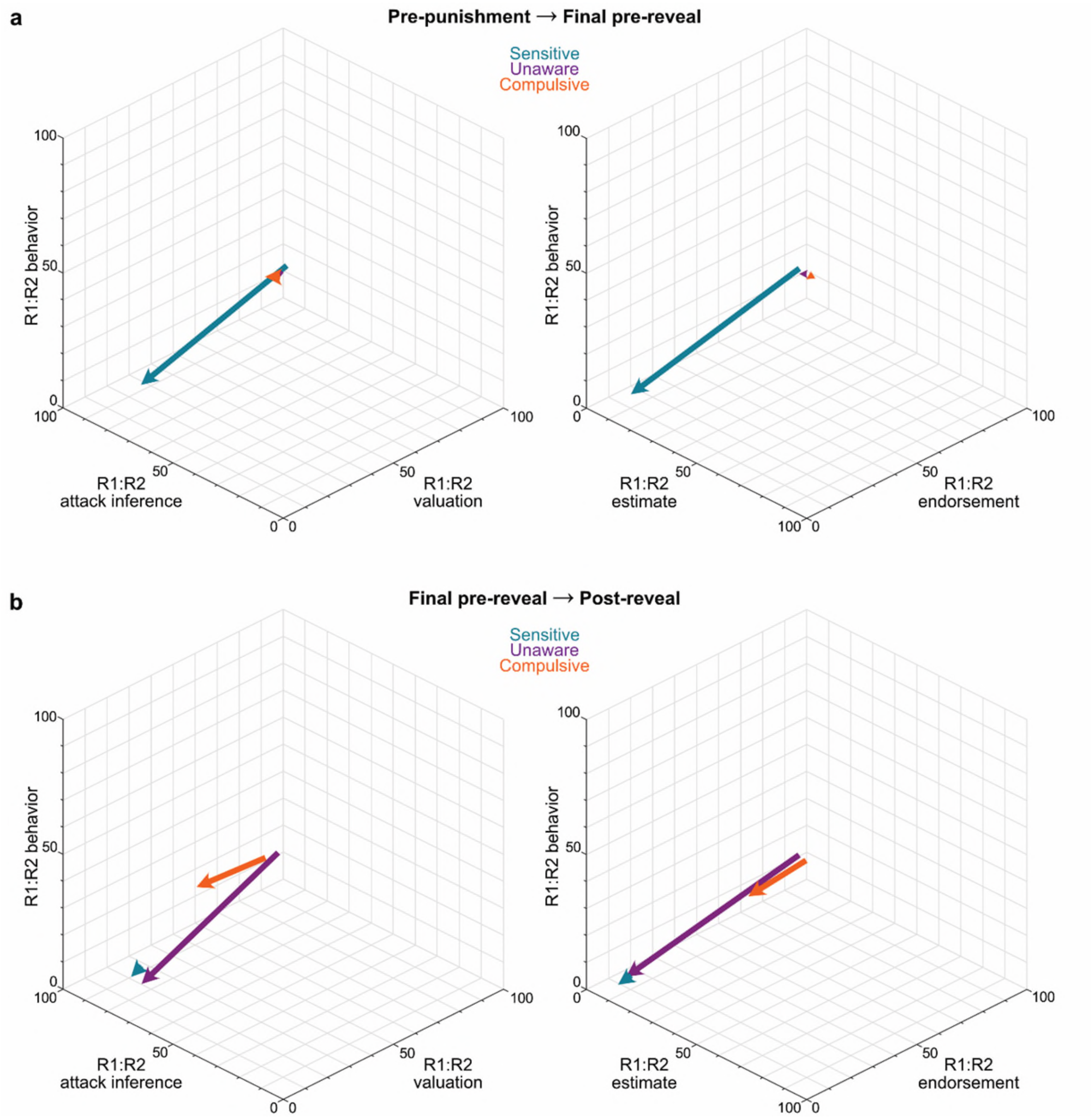

Figure S5. Cognitive-behavioral trajectories of clusters across task phases. (A) Mean Action-Attack inference bias, action value bias, and behavior preference [left panel], and endorsed preference, estimated preference, and behavior preference [right panel] per cluster from pre-punishment to final block of pre-reveal punishment (arrow tip). **(B)** Mean Action-Attack inference bias, action value bias, and behavior preference [left panel], and endorsed preference, estimated preference, and behavior preference [right panel] per cluster from final block of pre-reveal to post-reveal punishment (arrow tip).

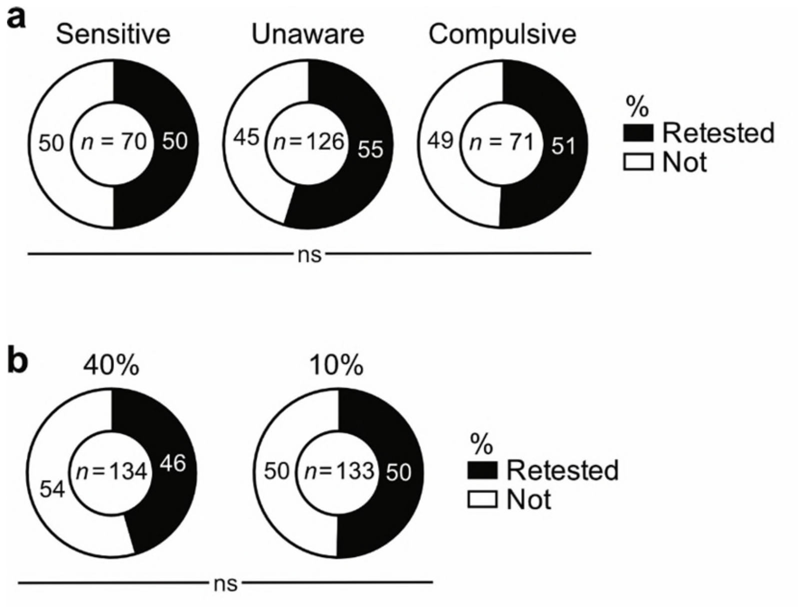

Figure S6. Test-retest retention of participants. (A) Proportion of original clusters (*N*=267) participants that underwent 6-month retest. There was no relationship between original cluster and who was retested ($\chi^2_{(2)}$=0.705, *p*=.703), indicating no selective attrition by cluster. **(B)** Proportion of original probability groups (*N*=267) that underwent 6-month retest. There was no relationship between group and who was retested ($\chi^2_{(1)}$=0.63, *p*=.427), indicating no selective attrition by group.

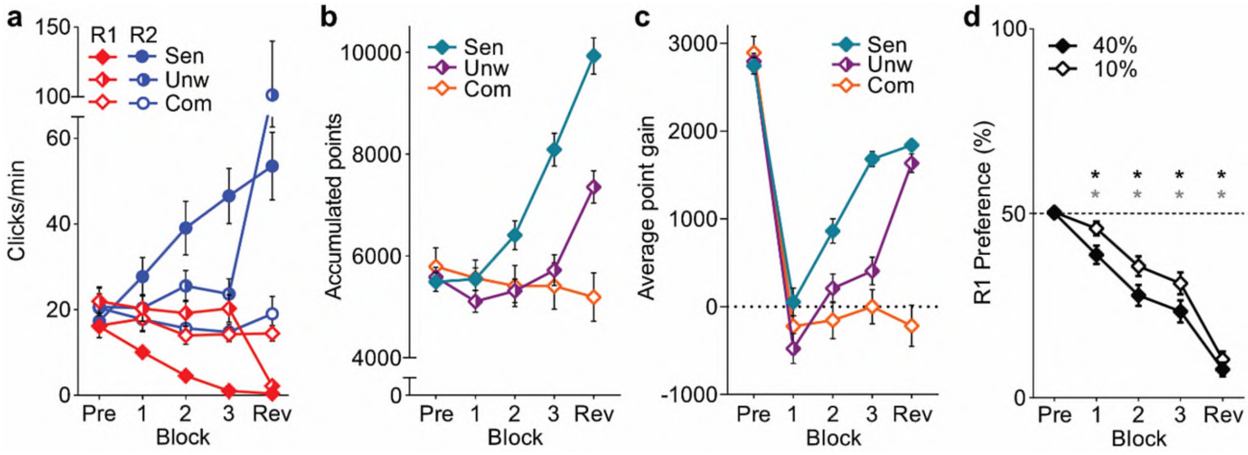

Figure S7. Additional task-related measures at retest. (A) Mean (±SEM) response rates during non-CS periods for Sensitive (Sen), Unaware (Unw), and Compulsive (Com) clusters. Clusters did not differ in their overall click rates across blocks (cluster [pre-reveal]: $F_{(2,125)}$=0.346, p=.709, η_p^2=.005 [90%CI: 0,.032]; block*cluster [pre-reveal]: $F_{(2,125)}$=0.069, p=.793, η_p^2=.001 [90%CI: 0,.005]), indicating similar effort expenditure to gain points. Clusters primarily differed in their response strategy before (action*block*cluster [pre-reveal]: $F_{(2,125)}$=8.149, p<.001, η_p^2=.115 [90%CI: .035,.198]) and after the reveal (action*block*cluster [reveal]: $F_{(2,125)}$=3.784, p=.025, η_p^2=.057 [90%CI: .004,.125]). Across pre-reveal blocks, Sensitive participants strongly reallocated effort away from R1 to R2 (action*block: $F_{(1,57)}$=48.02, p<.001,η_p^2=.457 [90%CI: .293,.571]), whereas Unawares modestly biased click rates (action: $F_{(1,51)}$=.049, p=.049, η_p^2=.074 [90%CI: 0,.046]; action*block: $F_{(1,51)}$=6.893, p=.011, η_p^2=.119 [90%CI: .015,.260]) and Compulsives did not (action: $F_{(1,17)}$=1.525, p=.234, η_p^2=.082 [90%CI: 0,.307]; action*block: $F_{(1,17)}$=1.439, p=.247, η_p^2=.078 [90%CI: 0, .301]). Following the reveal, Unawares significantly reallocated their responding (action: $F_{(1,51)}$=7.00, p=.011, η_p^2=.121 [90%CI: .016,.262]; action*block: $F_{(1,51)}$=6.102, p=.017, η_p^2=.107 [90%CI: .011,.245]), while Compulsives did not (action: $F_{(1,17)}$=2.837, p=.11, η_p^2=.143 [90%CI: 0,.374]; action*block: $F_{(1,17)}$=2.476, p=.134, η_p^2=.127 [90%CI: 0,.358]). **(B)** Mean (±SEM) accumulated points per cluster across retest blocks. Clusters differed in point accumulation across pre-reveal punishment (block*cluster: $F_{(2,125)}$=25.16, p<.001, η_p^2=.287 [90%CI: .174,.378]); only Sensitives gained points (block: $F_{(1,57)}$=87.54, p<.001,η_p^2=.606 [90%CI: .463,.692]), whereas other clusters did not (block [Unaware]: $F_{(1,51)}$=.423, p=.519,η_p^2=.008 [90%CI: 0,.089]; [Compulsive]: $F_{(1,17)}$=.779, p=.39,η_p^2=.044 [90%CI: 0,.252]). **(C)** Mean (±SEM) point gain across retest blocks. Clusters differed in point accumulation across pre-reveal (block*cluster: $F_{(2,125)}$=25.16, p<.001, η_p^2=.287 [90%CI: .174,.378]); only Sensitives gained points (block: $F_{(1,57)}$=87.54, p<.001, η_p^2=.606 [90%CI: .464,.692]), whereas Unawares (block: $F_{(1,51)}$=0.423, p=.519, η_p^2=.008 [90%CI: 0,.089]) and Compulsives (block: $F_{(1,17)}$=0.779, p=.39,η_p^2=.044 [90%CI: 0, .252]) did not. **(D)** Mean (±SEM) R1 preference per probability group across blocks. Lower probability (but higher severity) punishment caused less punishment avoidance than high probability (low severity) punishment (group [pre-reveal]: $F_{(1,126)}$=5.126, p=.025, η_p^2=.039 [90%CI: .003,.107]). N=128 participants.

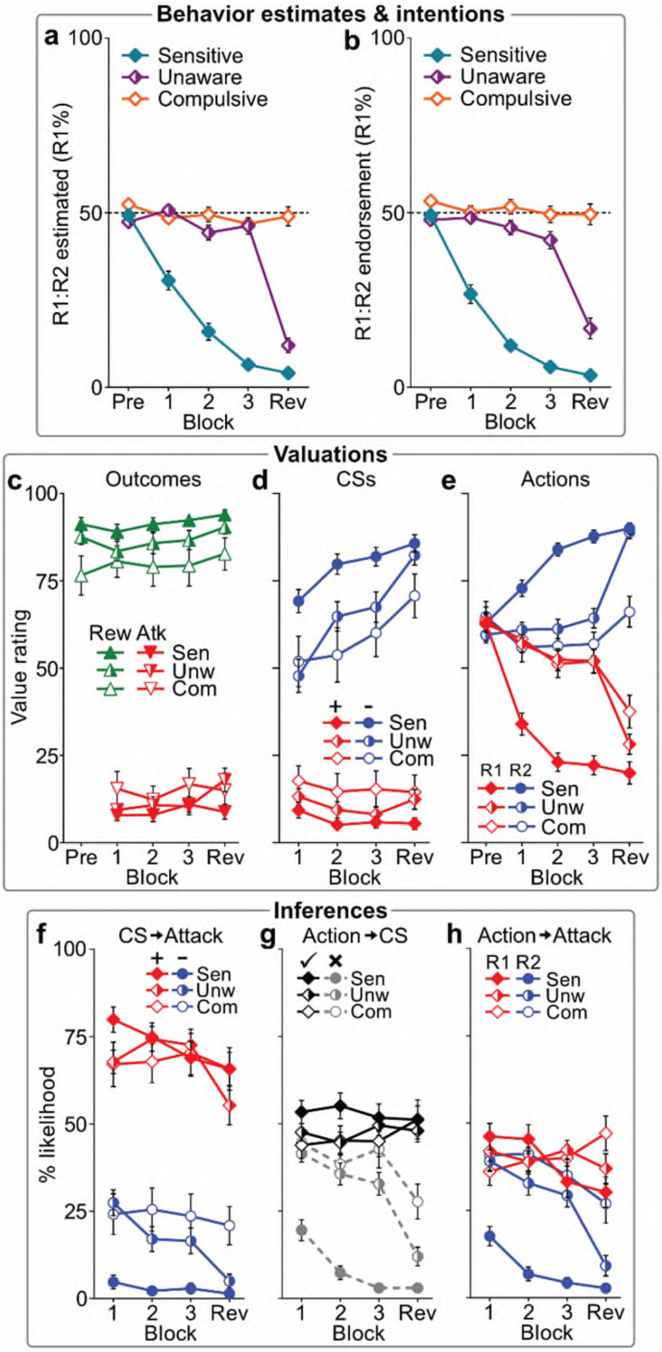

Figure S8. Psychological underpinnings of behavioral phenotypes at retest. [A-B] Mean (±SEM) post-block estimates of R1:R2 preference **(A)**, and post-block endorsed (perceived optimal) R1:R2 preference **(B)** per phenotype across blocks. Behavior estimates and endorsements matched actual behavior, suggesting behavior across phenotypes was deliberate, not habit-like. **[C-E]** Mean (±SEM) value ratings for point outcomes (reward [Rew], attack [Atk]), CSs (CS+, CS−), and actions (R1, R2) across blocks. **(C)** All clusters reported liking rewards (cluster: $F_{(2,125)}$=4.497, p=.013, η_p^2=.067 [90%CI: .008,.138]) and disliking attacks (cluster: $F_{(2,125)}$=1.631, p=.20, η_p^2=.025 [90%CI: 0,.076]). **(D)** All clusters rapidly disliked CS+ relative to CS− before contingency reveal (CS*block [pre-reveal]: $F_{(1,125)}$=28.64, p<.001, η_p^2=.186 [90%CI: .093,.283]; CS*block*cluster [pre-reveal]: $F_{(2,125)}$=1.723, p=.183, η_p^2=.027 [90%CI: 0,.079]). **(E)** Sensitives strongly discriminated action values before the reveal relative to other clusters (action*block*cluster [pre-reveal]: $F_{(2,125)}$=49.87, p<.001, η_p^2=.444 [90%CI: .332,.524]; action [Sensitive]: $F_{(1,57)}$=254.5, p<.001, η_p^2=.817 [90%CI: .740,.858]; [Unaware]: $F_{(1,51)}$=5.723, p=.02, η_p^2=.101 [90%CI: .009,.238]; [Compulsive]:

$F_{(1,17)}$=.855, p=.368, η_p^2=.048 [90%CI: 0,.258]). The reveal drove strong action revaluation for Unawares and, to a lesser extent, Compulsives (action*block*cluster: $F_{(2,125)}$=24.63, p<.001, η_p^2=.283 [90%CI: .170,.374]; [Unaware]: $F_{(1,51)}$=180.78, p<.001, η_p^2=.78 [90%CI: .682,.831]; [Compulsive]: $F_{(1,17)}$=11.338, p=.004, η_p^2=.40 [90%CI: .097,.589]). **[F-H]** Mean (±SEM) causal inferences per cluster across blocks. **(F)** All clusters were broadly aware that CS+, not CS-, led to Attack before the reveal (CS*block [pre-reveal]: $F_{(1,125)}$=0.541, p=.463, η_p^2=.004 [90%CI: 0,.042]; CS*block*cluster: $F_{(2,125)}$=3.889, p=.023, η_p^2=.059 [90%CI: .004,.127]). **(G)** Sensitives formed more accurate Action→CS (correct ✓ [R1→CS+ ; R2→CS-] over incorrect ✗ [R1→CS- ; R2→CS+]) inferences relative to other clusters before the reveal (correct*cluster: $F_{(2,125)}$=33.18, p<.001, η_p^2=.347 [90%CI: .232,.435]; correct [Sensitives]: $F_{(1,57)}$=149.3, p<.001, η_p^2=.724 [90%CI: .614,.785]; [Unaware]: $F_{(1,51)}$=10.45, p=.002, η_p^2=.17 [90%CI: .040,.316]; [Compulsive]: $F_{(1,17)}$=.589, p=.453, η_p^2=.034 [90%CI: 0,.234]). Contingency reveal drove inference updating in Unawares and Compulsives (correct*block*cluster: $F_{(2,125)}$=7.27, p=.001, η_p^2=.104 [90%CI: .028,.185]; correct*block [Unaware]: $F_{(1,51)}$=13.32, p<.001, η_p^2=.207 [90%CI: .063,.353]; [Compulsive]: $F_{(1,17)}$=5.285, p=.033, η_p^2=.241 [90%CI: .010,.461]). **(H)** Mean (±SEM) Action→Attack inferences per cluster across blocks. Clusters differed in their attribution of attacks to R1 vs R2 prior to reveal (action*cluster: $F_{(2,125)}$=83.96, p<.001, η_p^2=.278 [90%CI: .475,.638]); Sensitives strongly attributed attacks to R1 over R2 relative to other clusters (action [Sensitive]: $F_{(1,57)}$=94.57, p<.001, η_p^2=.596 [90%CI: .486,.706]; [Unaware]: $F_{(1,51)}$=7.754, p=.008, η_p^2=.132 [90%CI: .021,.274]; [Compulsive]: $F_{(1,17)}$=0.041, p=.842, η_p^2=.002 [90%CI: 0,.109]). Unawares (action: $F_{(1,51)}$=44.49, p<.001, η_p^2=.466 [90%CI: .292,.583]; action*block: $F_{(1,51)}$=4.687, p=.035, η_p^2=.084 [90%CI: .003,.217]) and Compulsives (action: $F_{(1,17)}$=10.62, p=.005, η_p^2=.385 [90%CI: .086,.578]; action*block: $F_{(1,17)}$=4.791, p=.043, η_p^2=.22 [90%CI: .004,.446]) exhibited greater Action-Attack awareness following the reveal. N=128 participants.

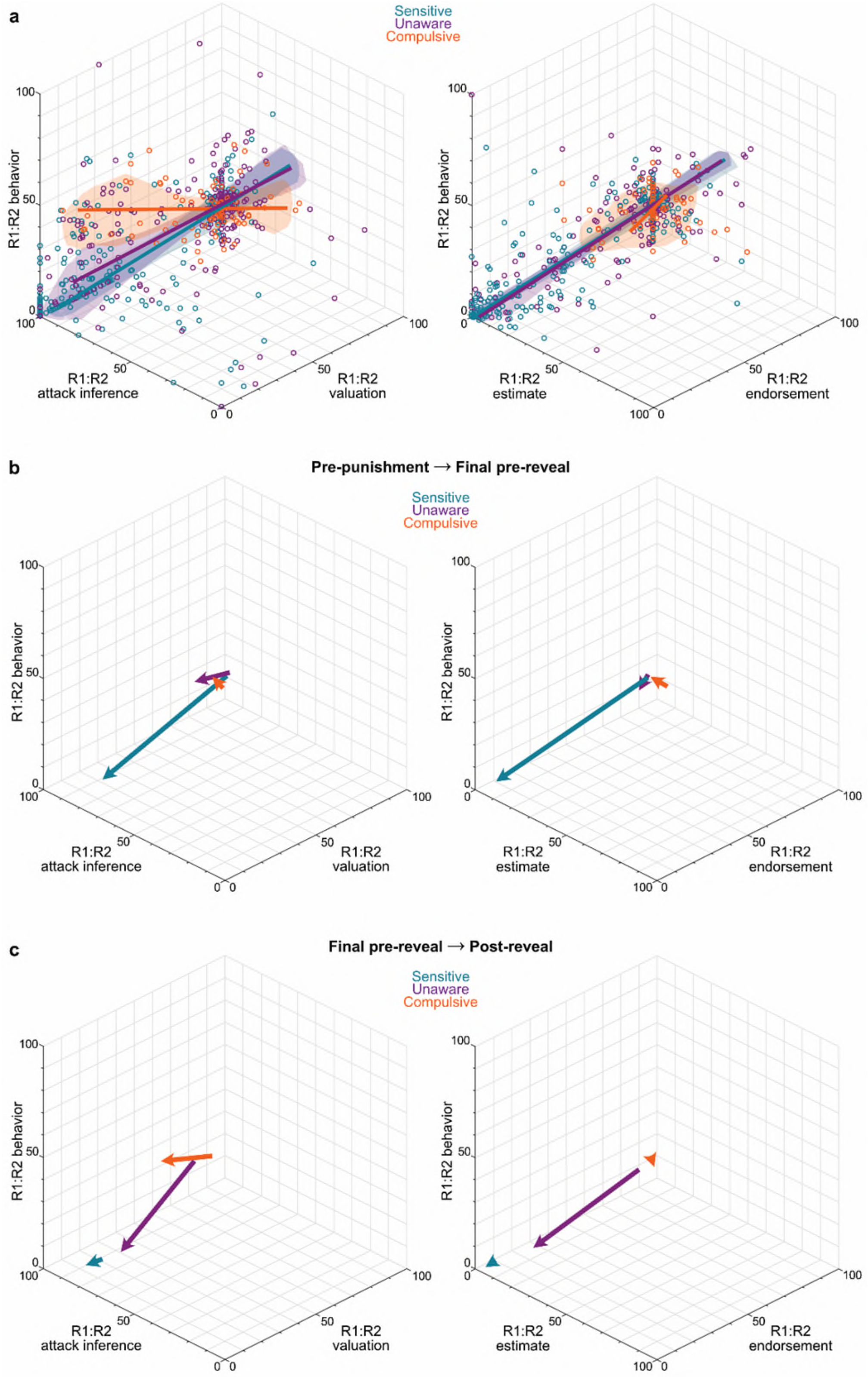

Figure S9. Cognitive-behavioral trajectories of phenotypes at retest. (A) True-score relationships between Action-Attack inference bias, action value bias, and behavior preference [left panel], and estimated preference, endorsed preference, and behavior preference [right panel]. Colored lines represent true-score relationships per cluster (determined via singular value decomposition). Shaded regions represent 3D confidence regions for true score relationships (determined via bootstrapping), and each dot represents

an individual's block score. Trajectory differences between clusters matched differences observed at initial test. **[B-C]** Cognitive-behavioral trajectories of clusters across task phases. **(B)** Mean Action-Attack inference bias, action value bias, and behavior preference [left panel], and endorsed preference, estimated preference, and behavior preference [right panel] per retest cluster from pre-punishment to final block of pre-reveal punishment (arrow tip). **(C)** Mean Action-Attack inference bias, action value bias, and behavior preference [left panel], and endorsed preference, estimated preference, and behavior preference [right panel] per retest cluster from final block of pre-reveal to post-reveal punishment (arrow tip).

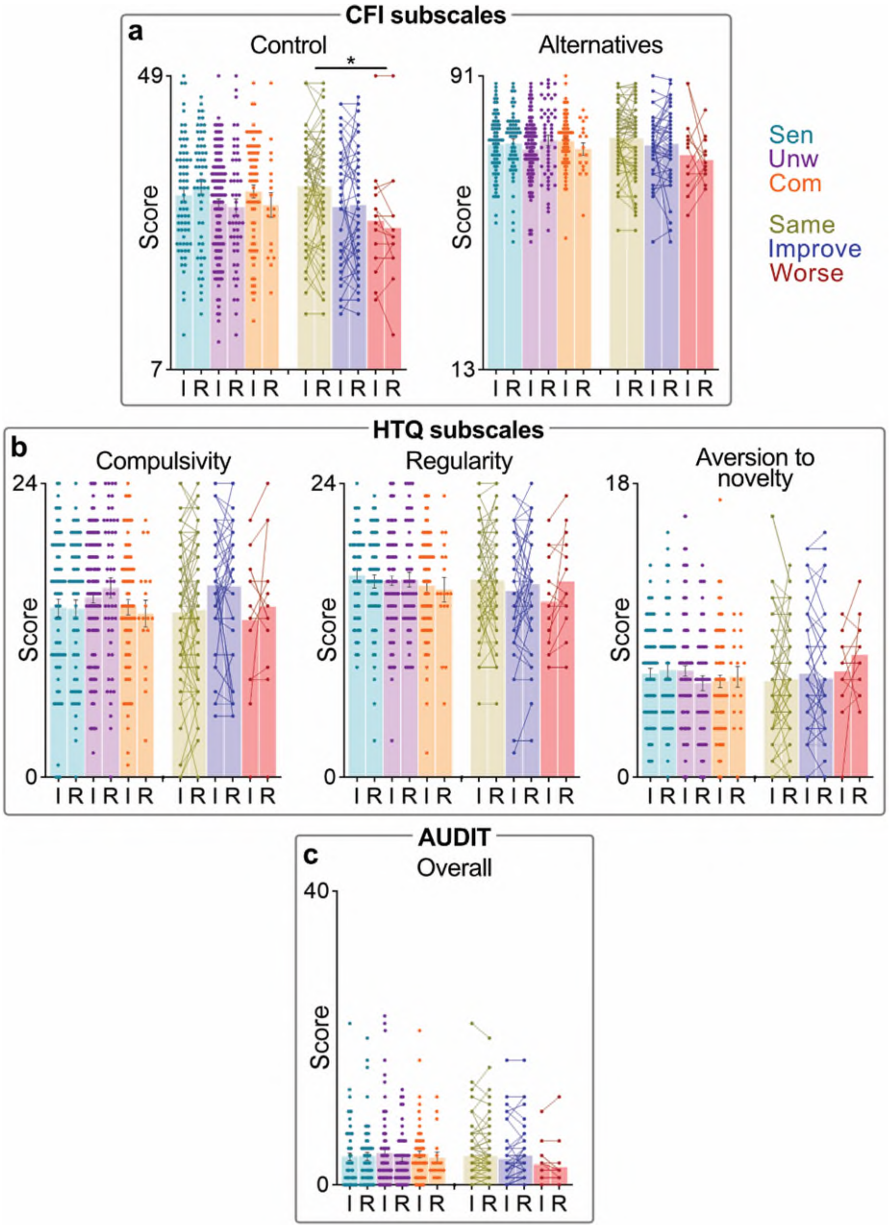

Figure S10. Self-reported traits at initial test (I) and retest (R). (A) Self-reported scores on Control [left panel; minimum score = 7] and Alternatives [right panel; minimum score = 13] subscales of the Cognitive Flexibility Inventory (CFI). Behavioral phenotypes (Sensitive [Sen], Unaware [Unw], Compulsive [Com]) did not differ on either CFI subscale at initial test (I [*N*=267]; all $F_{(2,264)}$≤2.038, *p*≥.132, η_p^2≤.015 [90%CI: 0,.043]) or retest (R [*N*=128]; $F_{(2,125)}$≤1.88, *p*≥.157, η_p^2≤.029 [90%CI: 0,.083]). Scores on Control subscale ($F_{(2,125)}$≥3.302, *p*≤.040, η_p^2≤.05 [90%CI: ≥.001, ≥.115]), but not Alternatives subscale ($F_{(2,125)}$≤2.376, *p*≥.097, η_p^2≤.037 [90%CI: 0,.095]), differed depending on whether participants maintained versus changed clusters across tests (*N*=128); participants that worsened across tests reported a lower sense of control than those that maintained their phenotype ([retest]: *p*=.04). **(B)** Self-reported scores on Compulsivity [left panel], Regularity [middle panel], and Aversion to novelty [right panel] subscales of the Habitual Tendencies Questionnaire (HTQ). Behavioral phenotypes did not significantly differ on any HTQ subscales ([initial clusters]: all $F_{(2,264)}$≤0.859, *p*≥.425, η_p^2≤.006 [90%CI: 0,.026]; [retest clusters] all $F_{(2,125)}$≤1.566, *p*≥.213, η_p^2≤.024 [90%CI: 0,.074]). Those that changed vs maintained their phenotype did significantly differ on any HTQ subscale scores (all $F_{(2,125)}$≤2.741, *p*≥.068, η_p^2≤.042 [90%CI: 0,.103]). **(C)** Self-reported scores on the Alcohol Use Disorders Identification Test (AUDIT). Behavioral phenotypes did not have significantly different AUDIT scores ([initial clusters]: $F_{(2,264)}$=0.283, *p*=.753, η_p^2=.002 [90%CI:

0,.014]; [retest clusters] $F_{(2,125)}$=0.037, p=.964 η_p^2<.001 [90%CI: 0,1]). Those that changed vs maintained their phenotype did not have significantly different AUDIT scores ($F_{(2,125)}$=1.053, p=.352, η_p^2=.017 [90%CI: 0,.060]).

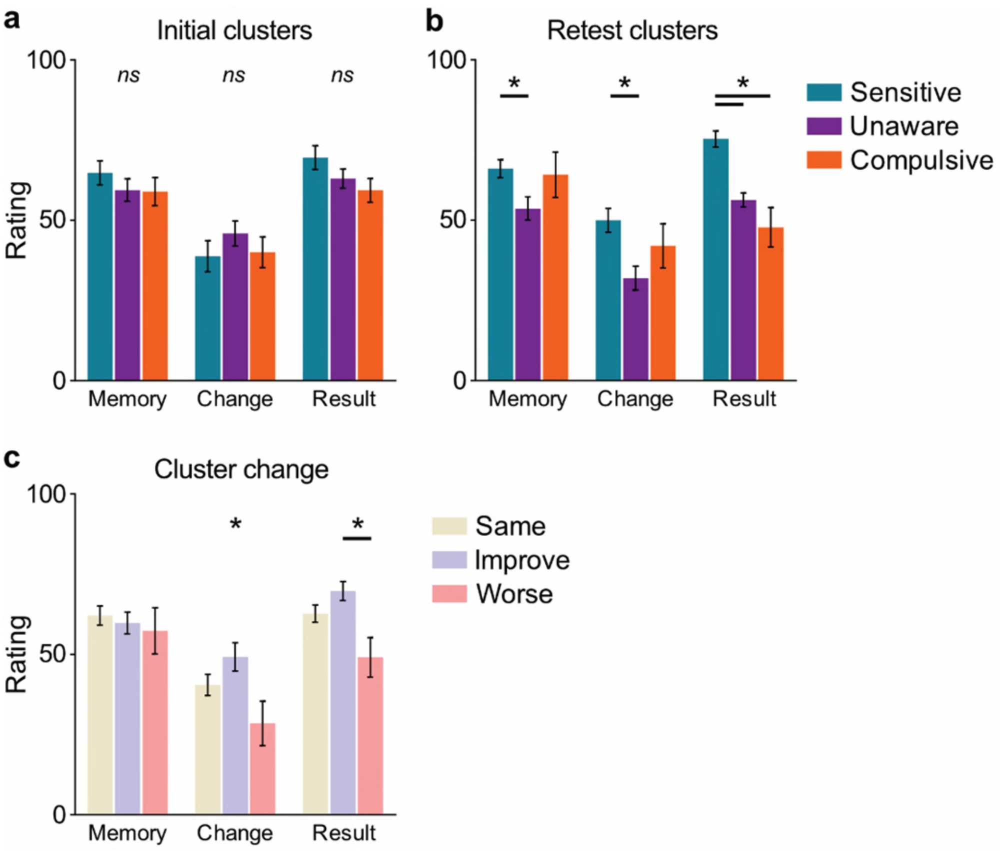

Figure S11. Post-retest reflections. Self-report ratings of how well participants remembered the initial test at retest (Memory), how much they chose to change their strategy across tests (Change), and whether they believed they achieved a better result at retest (Result). **(A)** Post-retest reflections by initial test cluster. Initial clusters did not differ on any measure (all $F_{(2,125)} \leq 1.91$, $p \geq .152$, $\eta_p^2 = .030$ [90%CI: 0,.083]). Crucially, this shows memory of the task did not depend on initial phenotype. **(B)** Post-retest reflections by retest cluster. Retest clusters differed in self-reported memory ($F_{(2,125)} = 3.643$, $p = .029$, $\eta_p^2 = .055$ [90%CI: .003,.122]), strategy change ($F_{(2,125)} = 5.668$, $p = .004$, $\eta_p^2 = .083$ [90%CI: .016,.159]), and results ($F_{(2,125)} = 19.43$, $p < .001$, $\eta_p^2 = .237$ [90%CI: .129,.329]). This was driven by Sensitives reporting better memory ($p = .029$) and more strategy change ($p = .003$) than Unawares, and Sensitives reporting better results relative to Unawares ($p < .001$) and Compulsives ($p < .001$). **(C)** Post-retest reflections by cluster change. Cluster stability did not significantly relate to memory of the task ($F_{(2,125)} = 0.259$, $p = .772$, $\eta_p^2 = .004$ [90%CI: 0,.026]), but did relate to strategy change ($F_{(2,125)} = 3.130$, $p = .047$, $\eta_p^2 = .048$ [90%CI: <.001,.111]), and result improvement ($F_{(2,125)} = 4.968$, $p = .008$, $\eta_p^2 = .074$ [90%CI: .011,.147]). Follow-up comparisons for strategy change were non-significant (Improve vs. Worse: $p = .055$; other $p \geq .300$). In line with actual performance, those with an improved avoidance phenotype reported better results than those with a worse phenotype ($p = .007$).

Table S1.

**1**<br>**Number of Clusters**	**2**<br>**Schwarz's Bayesian Criterion (BIC)**	**3**<br>**BIC Change**	**4**<br>**Ratio of BIC Changes**	**5**<br>**Ratio of Distance Measures**	**6**<br>**Mean Silhouette**
1	391.489				
2	254.375	-137.114	1	1.59	0.37916
**3**	**176.464**	**-77.911**	**0.568**	**3.008**	**0.58301**
4	165.476	-10.988	0.08	1.298	0.53535
5	162.141	-3.335	0.024	3.416	0.55805
6	176.97	14.83	-0.108	1.042	
7	192.102	15.131	-0.11	1.011	
8	207.31	15.209	-0.111	1.9	
9	225.901	18.59	-0.136	1.45	
10	245.657	19.756	-0.144	1.138	
11	265.728	20.071	-0.146	1.37	
12	286.414	20.686	-0.151	1.064	
13	307.201	20.786	-0.152	1.135	
14	328.173	20.972	-0.153	1.17	
15	349.345	21.172	-0.154	1.096	

Table S1. Outputs for TwoStep clustering solution. Auto-clustering using the TwoStep algorithm determined 3 clusters as optimal. Follow-up silhouette analyses confirmed that the 3-cluster solution had the lowest average silhouette value.
