# Supplementary material for: Causal inference and cognitive-behavioral integration deficits drive stable variation in human punishment sensitivity
Source: Commun Psychol. 2025 Jul 9;3:103. doi: 10.1038/s44271-025-00284-9 (PMC12241639; doi:10.1038/s44271-025-00284-9)
Supplement: Supplementary file 3 — Reporting summary [file 44271_2025_284_MOESM3_ESM.pdf]

nature portfolio

Corresponding author(s): Philip Jean-Richard-dit-Bressel

Last updated by author(s): Jun 5, 2025

Reporting Summary

Nature Portfolio wishes to improve the reproducibility of the work that we publish. This form provides structure for consistency and transparency in reporting. For further information on Nature Portfolio policies, see our Editorial Policies and the Editorial Policy Checklist.

Statistics

For all statistical analyses, confirm that the following items are present in the figure legend, table legend, main text, or Methods section.

n/a	Confirmed	
☐	☒	The exact sample size (*n*) for each experimental group/condition, given as a discrete number and unit of measurement
☐	☒	A statement on whether measurements were taken from distinct samples or whether the same sample was measured repeatedly
☐	☒	The statistical test(s) used AND whether they are one- or two-sided<br>*Only common tests should be described solely by name; describe more complex techniques in the Methods section.*
☐	☒	A description of all covariates tested
☐	☒	A description of any assumptions or corrections, such as tests of normality and adjustment for multiple comparisons
☐	☒	A full description of the statistical parameters including central tendency (e.g. means) or other basic estimates (e.g. regression coefficient) AND variation (e.g. standard deviation) or associated estimates of uncertainty (e.g. confidence intervals)
☐	☒	For null hypothesis testing, the test statistic (e.g. *F*, *t*, *r*) with confidence intervals, effect sizes, degrees of freedom and *P* value noted<br>*Give P values as exact values whenever suitable.*
☒	☐	For Bayesian analysis, information on the choice of priors and Markov chain Monte Carlo settings
☐	☒	For hierarchical and complex designs, identification of the appropriate level for tests and full reporting of outcomes
☐	☒	Estimates of effect sizes (e.g. Cohen's *d*, Pearson's *r*), indicating how they were calculated

Our web collection on statistics for biologists contains articles on many of the points above.

Software and code

Policy information about availability of computer code

Data collection	Data was collected via Prolific using scripts programmed with the jsPsych library. As noted in manuscript, all experiment code is posted in https://github.com/philjrdb/HCP-Test-Retest and https://zenodo.org/records/155819599.
Data analysis	As indicated in the manuscript, all data was extracted and preprocessed using custom MATLAB scripts (made available at https://github.com/philjrdb/HCP-Test-Retest). Clustering, chi-square, ANOVAs, t-tests, and stepwise logistic regression were performed in SPSS (version 29.0). Singular Value Decomposition was performed in MATLAB (R2022b).

For manuscripts utilizing custom algorithms or software that are central to the research but not yet described in published literature, software must be made available to editors and reviewers. We strongly encourage code deposition in a community repository (e.g. GitHub). See the Nature Portfolio guidelines for submitting code & software for further information.

Data

Policy information about availability of data

All manuscripts must include a data availability statement. This statement should provide the following information, where applicable:

- Accession codes, unique identifiers, or web links for publicly available datasets
- A description of any restrictions on data availability
- For clinical datasets or third party data, please ensure that the statement adheres to our policy

All de-identified experiment data is provided at https://osf.io/ju35h/.

Research involving human participants, their data, or biological material

Policy information about studies with human participants or human data. See also policy information about sex, gender (identity/presentation), and sexual orientation and race, ethnicity and racism.

Reporting on sex and gender	Data was analyzed by self-reported gender (118 female, 143 male, 6 other).
Reporting on race, ethnicity, or other socially relevant groupings	Participants were a general population sample from 24 OECD countries. Data on sexual orientation, race, and ethnicity were not collected.
Population characteristics	Participant were aged 18-63 years (mean age = 32.17, SD = 10.74). Age distribution is reported in Figure S1.
Recruitment	Participants were recruited via Prolific (online research platform). Self-reported fluency in English was a selection criterion.
Ethics oversight	The study was approved by UNSW Human Research Ethics Advisory Panel C (HREAP-C #3385).

Note that full information on the approval of the study protocol must also be provided in the manuscript.

Field-specific reporting

Please select the one below that is the best fit for your research. If you are not sure, read the appropriate sections before making your selection.

☐ Life sciences ☒ Behavioural & social sciences ☐ Ecological, evolutionary & environmental sciences

For a reference copy of the document with all sections, see nature.com/documents/nr-reporting-summary-flat.pdf

Behavioural & social sciences study design

All studies must disclose on these points even when the disclosure is negative.

Study description	Quantitative experimental
Research sample	General population sample recruited from Prolific
Sampling strategy	Participants were randomly sampled from Prolific. Participants that passed all engagement checks in the initial test were invited to participate in the retest experiment (immediately prior to retest data collection).
Data collection	Data was collected using scripts programmed with the jsPsych library (https://github.com/philjrdb/HCP-Test-Retest).
Timing	Initial test data was collected in Jan 2023, and retest data was collected in Jul 2023.
Data exclusions	Participants were excluded from analyses if they failed either of two engagement checks: 1) failing to give correct responses to two catch questions embedded in the questionnaire battery (see Self-reported trait questionnaires for details); or 2) answering post-block measures too quickly or slowly (<0.8s or >30s per question, averaged per page). These criteria were pre-established.
Non-participation	No participants declined participation or dropped out during initial test. Participation at retest was optional; 128 (~50%) of initial included participants were included at retest - no selective attrition of participants was observed.
Randomization	Participants were randomly allocated to probability groups. Retested participants were allocated to the same probability groups as initial test.

Reporting for specific materials, systems and methods

We require information from authors about some types of materials, experimental systems and methods used in many studies. Here, indicate whether each material, system or method listed is relevant to your study. If you are not sure if a list item applies to your research, read the appropriate section before selecting a response.

Materials & experimental systems

n/a	Involved in the study
☒	☐ Antibodies
☒	☐ Eukaryotic cell lines
☒	☐ Palaeontology and archaeology
☒	☐ Animals and other organisms
☒	☐ Clinical data
☒	☐ Dual use research of concern
☒	☐ Plants

Methods

n/a	Involved in the study
☒	☐ ChIP-seq
☒	☐ Flow cytometry
☒	☐ MRI-based neuroimaging

Plants

Seed stocks	N/A
Novel plant genotypes	N/A
Authentication	N/A
